# Screening cluster A and cluster B personality disorders in Chinese high school students

**DOI:** 10.1186/1471-244X-13-116

**Published:** 2013-04-17

**Authors:** Yuping Wang, Xiongzhao Zhu, Lin Cai, Qin Wang, Mengcheng Wang, Jinyao Yi, Shuqiao Yao

**Affiliations:** 1 Medical Psychological Institute, Second Xiangya Hospital, Central South University, Changsha, 410011, P. R. China; 2 Hunan Province Technology Institute of Psychiatry, Central South University, Changsha, 410011, P. R. China; 3 Key Laboratory of Psychiatry and Mental Health of Hunan Province, Central South University, Changsha, 410011, P. R. China

**Keywords:** Personality disorders, High school student, Cross-sectional study

## Abstract

**Background:**

Personality disorders (PDs) during adolescence may, in addition to increasing risk for violent behaviors and suicide, also increase risk for elevated PD traits in adulthood. The aim of this study was to explore the prevalence of Cluster A and Cluster B PD traits and their relationships to demographic variables in Chinese high school students.

**Methods:**

A cohort of 3,552 students from eight high schools completed the Personality Diagnostic Questionnaire-4+ (PDQ-4+) and MacArthur Scale of Subjective Social Status-youth version (SSSy) questionnaires.

**Results:**

Boys scored higher than girls on the paranoid, schizotypal, antisocial, and narcissistic PDs. Freshmen and sophomores scored higher than juniors on schizoid, borderline, and antisocial PDs. Children in single-child families scored higher than nonsingletons on the paranoid and antisocial PDs. Students from single-parent households scored higher than students from double-parent households on the schizotypal and antisocial PDs, and students with remarried parents scored higher than students from double-parent households on the borderline and antisocial PDs. Students who had low perception of social status in the society ladder scored higher than those with a high perceived status on the schizoid and borderline PDs, but scored lower on the histrionic PD; students with a low subjective social status in the school community ladder scored higher scores than those with a high perceived status on the paranoid, schizoid, borderline, and antisocial PDs, but scored lower on the histrionic PD.

**Conclusions:**

Gender, grade, family structure, and subjective social status may affect the development of PDs. Longitudinal studies and studies of the full scope of PDs are needed to fully elucidate the impact of demographic variables on PD prevalence rates in adolescence and adulthood.

## Background

The *DSM-IV-TR* (2000) describes personality disorders (PDs) as “inflexible and maladaptive personality characteristics [that] cause significant functional impairment or subjective distress” and as having an “enduring pattern of perceiving, related to, and thinking about the environment and oneself that are exhibited in a wide range of social and personal contexts”[1]. PDs are thought to be chronic rather than intermittent, originating in childhood and continuing throughout adulthood, pervading every aspect of a person’s life [2].

According to traditional personality theory, people’s personalities become relatively stable after about 18 years of age. Accordingly, most previous studies have focused on adult samples, and only adults can be diagnosed definitively as having a PD in major psychiatric diagnostic systems. Recently, there has been a growing interest in researching PDs in adolescents, especially during early adolescence, the developmental stage that is considered a critical phase in the onset and development of PDs. Golombek et al. found that 46% of the 13-year-old secondary school students in their study sample showed some degree of personality dysfunction, enough to center an axis II diagnosis [3]. Similarly, Korenblum and colleagues reported that 42% of the adolescents in their nonclinical groups showed symptoms of personality disorders to varying degrees, with at least 33% meeting the diagnostic criteria for a PD [4]. Epidemiological surveys of adolescent PDs in community juvenile [5], clinical outpatient [6,7], and juvenile offender [8] populations have since been carried out.

PDs are not rare in the general adolescent population, and researchers have come to realize the clinical importance of an abnormal personality in early adolescence. Several studies have indicated that PDs are often associated with violent and risky behavior in adolescents. Johnson et al. found that adolescents with a greater number of DSM-IV cluster A or cluster B PD symptoms were more likely than other adolescents in the community to resort to violent acts during adolescence and in early adulthood [5]. Moran and his colleagues reported that co-morbid PD is independently associated with an increased risk of violent behavior in psychosis [9]. Brent et al. found that having a PD was a critical risk factor for suicide completion [10].

PD in adolescence may have profound impact on personality traits in adulthood. Kasen et al. reported that having a PD in adolescence may increase the risk of later exhibiting a PD in adulthood in the same cluster [11]. Likewise, adolescents with PDs have been reported to show elevated personality disorder traits during early adulthood [12]. Therefore, it would be prudent to identify emerging personality pathologies before adulthood. Moreover, improving our understanding of PD precursors in adolescence may help to reduce the risk of subsequent exasperation of the condition. In China, in particular, researchers have focused on college aged populations, and there are no large-sample studies of adolescents.

This study was part of a larger research project examining risk behaviors and their related factors in Chinese adolescents. Because cluster A and cluster B PD symptoms have been shown to be closely related to violent acts during adolescence [5], we focused our study on cluster A and cluster B PDs only. The aim of this study was to explore the prevalence of Cluster A and Cluster B PD traits and their relationships to demographic variables in a large sample of Chinese high school students.

## Methods

### Procedure

The experimental procedures of this study were approved by the Ethics Committee of Central South University. Four thousand written informed consent forms explaining the aim and procedures of this study were sent to high school students’ guardians (mostly parents), and 3594 signed forms were returned (participation rate 89.9%). Subsequently, 3594 students also signed written informed consent forms and completed the Personality Diagnostic Questionnaire-4+ (PDQ-4+), the MacArthur Scale of Subjective Social Status-youth version (SSSy), and a general questionnaire that collected information about demographic variables. Forty two participants were excluded for they did not fill out the surveys at least one item, leaving an effective sample size of 3552, therefore, the effective rate of response was 98.8%.

### Participants

A total of 3,552 high school students (50.4% males) were enrolled from eight high schools located across seven geographic districts in China, encompassing a generally representative sample of Chinese high school students with regard to socioeconomic status and most demographic variables. The mean age of the students was 16.62 years (SD = 1.11, range = 14–20). The sample included 39.8% freshmen, 35.3% sophomores, and 24.9% juniors. Most of the participants (61.0%) were singletons (the only children in their families). A large majority of the children (92.5%) were of Han Chinese ethnicity, and 7.5% were ethnic minorities (Table 1).

**Table 1:** The sociodemographic characteristics of the samples, N (%)

		**Male**	**Female**	**Total**
Gender		1790(50.4%)	1762(49.6%)	3552(100%)
Age	≤15	251(13.6%)	297(16.9%)	558(15.6%)
	16	595(33.2%)	547(31.0%)	1142(32.2%)
	17	537(30.0%)	540(30.6%)	1077(30.3%)
	18	308(17.2%)	312(17.7%)	620(17.5%)
	≥19	89(5.0%)	66(3.8%)	155(4.4%)
Grade ^a^	Freshman	685(38.3%)	728(41.3%)	1413(39.8%)
	Sophomore	680(38.0%)	574(32.6%)	1254(35.3%)
	Junior	425(23.7%)	460(26.1%)	885(24.9%)
Single-child status ^b^	Singletons	1168(65.3%)	999(56.7%)	2167(61.0%)
	Nonsingletons	622(34.7%)	763(43.3%)	1385(39.0%)
Parents’ marital status	Married	1583(88.4%)	1565(88.8%)	3148(88.6%)
	Divorced	142(7.9%)	143(8.1%)	285(8.0%)
	Remarried	65(3.6%)	54(3.1%)	119(3.4%)

Notes: ^a^ Gender was not equivalent in grade (*χ*2 = 11.433, df = 2, p = 0.003); ^b^ Gender was not equivalent in single-child status (*χ*2 = 27.315, df = 1, p < 0.001).

### Measures

#### General questionnaire

The general questionnaire collected data on the following parameters: age, gender, ethnicity, singleton or having siblings, and parents’ marital status.

#### PDQ-4+

The Personality Diagnostic Questionnaire-4+ (PDQ-4+) is the most recent version of a self-report measure developed by Hyler to assess DSM-IV personality disorders [13]. The PDQ-4+ closely fits with DSM personality disorder descriptors; each item reflects precisely a single DSM-IV diagnostic criterion. As a result, it has been strongly recommended for brief screenings [14]. The PDQ-4+ is a forced-choice, 99-item questionnaire designed to assess the 10 PDs indicated in the DSM-IV-TR, namely paranoid PD, schizoid PD, schizotypal PD, antisocial PD, borderline PD, histrionic PD, narcissistic PD, avoidant PD, dependent PD, and obsessive-compulsive PD, as well as passive aggressive (negativistic) PD and depressive PD. It includes two validity scales to detect fake responding: the Too Good (TG) and Suspect Questionnaire (SQ) scales [15]. The PDQ-4+ total score serves as a general index of a personality disturbance of any kind. The psychometric properties of the PDQ-4+ have been shown to be satisfactory, both in the questionnaire’s original version [13] and in its adaptation to other languages and cultures [16-18]. The PDQ-4+, which was translated and adapted for use in China by Yang Jian [18], has good alpha coefficients, ranging from .49 (passive-aggressive) to .72 (depressive), and retest reliability, ranging from .48 to .79, and has been widely used in Chinese population samples [18-22]. In this study, we focused on the seven personality disorders in cluster A (paranoid, schizoid, and schizotypal) and cluster B (antisocial, borderline, histrionic, narcissistic), which are closely related to violent behaviors (77 items).

#### SSSy

The MacArthur Scale of Subjective Social Status-youth version (SSSy) [23] was used to assess current subjective perceptions of social status among students. This instrument is a self-anchoring scale in the form of two 10-rung ladders (social ladder and school community ladder) and has excellent 2-month test-retest reliability [23]. SSSy scores were recorded as a dichotomic variable, with scores in the range of 1–5 being recorded as 1 (low subjective social status), and scores in the range of 6–10 being recorded as 2 (high subjective social status). The SSSy translated and adapted for use in China by Hu Muli, has good reliability and validity [24].

#### Statistical analysis

Descriptive analyses, independent-sample t-tests, and analyses of co-variance (ANCOVAs) were performed with SPSS 15.0 software. Independent-sample t-tests were performed to examine the influence of gender. ANCOVAs were performed to examine the influence of grade, single-child status, parents’ marital status, and subjective social status with gender and age as covariates. The data are reported as mean values with standard deviations (SDs). Comparisons that yielded *p* values < 0.05 were considered significant.

## Results

### Descriptive statistics

**Figure 1:**
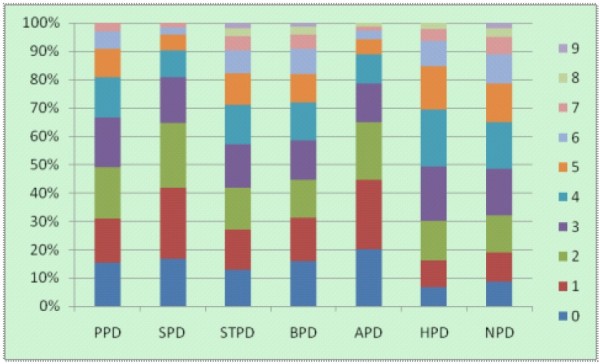
The score distributions of cluster A and cluster B PDs.

The sociodemographic characteristics of the sample are presented in Table 1. Of the 3552 high school students enrolled in this study, 1790 (50.4%) were male (*χ*^2^ = 0.221, df = 1, *p* = 0.638). The boys had a mean age of 16.65 years (SD = 1.109) and the girls had a mean age of 16.60 (SD = 1.110; t = 1.355, p = 0.176). Gender distribution varied by grade (*χ*^2^ = 11.433, df = 2, p = 0.003), and single-child status (*χ*2 = 27.315, df = 1, p < 0.001). The frequency distributions of cluster A and cluster B PD scores are presented in Table 2, and the score distributions of cluster A and B PDs were presented in Figure 1.

**Table 2:** The frequency distribution of cluster A and cluster B PD scores, N (%)

**Subscales scores of subscales**	**0**	**1**	**2**	**3**	**4**	**5**	**6**	**7**	**8**	**9**
PPD	549(15.5%)	546(15.4%)	642(18.1%)	630(17.7%)	505(14.2%)	357(10.1%)	213(6.0%)	110(3.1%)		
SPD	591(16.6%)	890(25.1%)	818(23.0%)	570(16.0%)	336(9.5%)	196(5.5%)	90(2.5%)	61(1.7%)		
STPD	455(12.8%)	505(14.2%)	525(14.8%)	546(15.4%)	489(13.8%)	399(11.2%)	291(8.2%)	176(5.0%)	94(2.6%)	72(2.0%)
BPD	566(15.9%)	544(15.3%)	476(13.4%)	489(13.8%)	475(13.4%)	361(10.2%)	313(8.8%)	182(5.1%)	99(2.8%)	47(1.3%)
APD	709(20.0%)	876(24.7%)	719(20.2%)	490(13.8%)	361(10.2%)	194(5.5%)	107(3.0%)	51(1.4%)	45(1.3%)	
HPD	238(6.7%)	340(9.6%)	495(13.9%)	674(19.0%)	719(20.2%)	545(15.3%)	316(8.9%)	146(4.1%)	79(2.2%)	
NPD	310(8.7%)	363(10.2%)	467(13.1%)	577(16.2%)	584(16.4%)	487(13.7%)	371(10.4%)	216(6.1%)	110(3.1%)	67(1.9%)

Notes: *PPD*: Paranoid personality disorder; *SPD*: Schizoid personality disorder; *STPD*: Schizotypal personality disorder; *BPD*: Borderline personality disorder; *APD*: Antisocial personality disorder; *HPD*: Histrionic personality disorder; *NPD*: Narcissistic personality disorder; Similarly hereinafter.

### Influence of gender and grade on PD traits

Boys scored higher than girls on the paranoid, schizotypal, antisocial, and narcissistic PDs with a weak effect size. As a result of gender distribution not being equivalent across the grades, we found an effect of grade on PDQ-4+ scores with gender as a covariate. Freshmen and sophomores scored higher than juniors on the schizoid, borderline, and antisocial PDs; freshman and sophomore scores did not differ from each other (Table 3).

**Table 3:** Comparison of cluster A and B PD scores on gender, and among grades with gender as covariate

	**Total n = 3552**	**Gender**				**Grades**			
		**Male n = 1790**	**Female n = 1762**	**t**	**Cohen’s d**	**Freshman n = 1413**	**Sophomore n = 1254**	**Junior n = 885**	**F**
PPD	2.70 ± 1.92	2.81 ± 1.93	2.58 ± 1.90	3.679*	0.120	2.75 ± 1.92	2.69 ± 1.93	2.61 ± 1.89	1.320
SPD	2.12 ± 1.67	2.16 ± 1.80	2.08 ± 1.51	1.551	0.048	2.21 ± 1.70^a^	2.17 ± 1.70^b^	1.91 ± 1.53	9.025*
STPD	3.24 ± 2.31	3.52 ± 2.37	2.96 ± 2.21	7.341*	0.244	3.32 ± 2.33	3.26 ± 2.30	3.09 ± 2.28	2.692
BPD	3.11 ± 2.34	3.11 ± 2.38	3.10 ± 2.30	.086	0.004	3.25 ± 2.41^a^	3.14 ± 2.32^b^	2.83 ± 2.25	8.935*
APD	2.13 ± 1.834	2.38 ± 1.98	1.86 ± 1.63	8.604*	0.287	2.21 ± 1.89^a^	2.25 ± 1.88^b^	1.82 ± 1.62	15.231*
HPD	3.52 ± 1.92	3.55 ± 2.01	3.49 ± 1.82	.826	0.031	3.54 ± 1.95	3.52 ± 1.92	3.49 ± 1.87	0.199
NPD	3.67 ± 2.23	3.76 ± 2.31	3.57 ± 2.14	2.650*	0.085	3.75 ± 2.27	3.61 ± 2.24	3.61 ± 2.14	1.826

Notes: *P < 0.05; ^a^ Compared with Junior *P* < 0.05; ^b^ Compared with Junior *P* < 0.05.

### Influence of family structure on PD traits

Family structure was found to be related to the incidence of PD traits. Singleton youths scored higher than kids with siblings on the paranoid and antisocial PDs with gender and age as covariates, but the effect size is weak. After controlling for gender and age, we found that children in single-parent families had significantly higher scores than children from double-parent families on the schizotypal and antisocial PDs, while kids from remarried families scored higher than those from double-parent families on the borderline and antisocial PDs (Table 4).

**Table 4:** Comparison of cluster A and B PD scores on single-child status, and among parents’ marital status with gender and age as covariates

	**Singletons and nonsingletons**				**Parents’ Marriage**			
	**Singletons n = 2167**	**Nonsingletons n = 1385**	**F**	**Cohen’s d**	**Married n = 3148**	**Divorced n = 285**	**Remarried n = 119**	**F**
PPD	2.76 ± 1.94	2.59 ± 1.88	4.664*	0.089	2.68 ± 1.92	2.82 ± 1.95	2.80 ± 1.87	0.438
SPD	2.17 ± 1.71	2.04 ± 1.59	3.305	0.079	2.10 ± 1.66	2.21 ± 1.64	2.39 ± 1.78	1.981
STPD	3.29 ± 2.36	3.17 ± 2.22	0.270	0.052	3.21 ± 2.30 ^a^	3.53 ± 2.32	3.50 ± 2.29	3.258*
BPD	3.15 ± 2.37	3.04 ± 2.30	0.684	0.047	3.07 ± 2.35 ^b^	3.32 ± 2.18	3.58 ± 2.49	3.889*
APD	2.22 ± 1.89	1.98 ± 1.73	7.704*	0.132	2.10 ± 1.84 ^a,b^	2.33 ± 1.74	2.44 ± 1.91	3.563*
HPD	3.55 ± 1.95	3.47 ± 1.87	1.222	0.042	3.50 ± 1.92	3.67 ± 1.87	3.65 ± 1.95	1.218
NPD	3.67 ± 2.25	3.66 ± 2.19	0.065	0.005	3.64 ± 2.22	3.87 ± 2.21	3.85 ± 2.32	1.784

Notes: *P < 0.05; ^a^ Compared with Divorced *P* < 0.05; ^b^ Compared with Remarried *P* < 0.05.

### Influence of perceived social status on PD traits

With gender and age as covariates, we found that students who had a low subjective perception of social status in the society ladder of the SSSy scored higher on the schizoid and borderline PDs than those who had a high perceived status, but had a significantly lower score on the histrionic PD. However, all the effect sizes are weak (Table 5). Students who had a low perceived social status in the school community ladder of the SSSy scored higher on the paranoid, schizoid, borderline, and antisocial PDs than students who had a high perceived status, but scored lower on the histrionic PD, while all the effect sizes are weak (Table 5).

**Table 5:** Comparison of cluster A and B PD scores on subjective social status with gender and age as covariates

	**Subjective social status in society**				**Subjective social status in school community**			
	**Low**	**High**	**F**	**Cohen’s d**	**Low**	**High**	**F**	**Cohen’s d**
PPD	2.75 ± 1.94	2.64 ± 1.89	3.215	0.057	2.85 ± 2.01	2.63 ± 1.87	9.266*	0.113
SPD	2.19 ± 1.65	2.05 ± 1.68	5.945*	0.084	2.29 ± 1.71	2.04 ± 1.64	16.421*	0.150
STPD	3.30 ± 2.30	3.19 ± 2.31	1.960	0.048	3.34 ± 2.36	3.20 ± 2.28	1.803	0.060
BPD	3.26 ± 2.36	2.95 ± 2.32	16.538*	0.132	3.45 ± 2.45	2.95 ± 2.28	34.048*	0.211
APD	2.17 ± 1.85	2.08 ± 1.82	1.894	0.049	2.32 ± 1.94	2.04 ± 1.77	14.690*	0.151
HPD	3.44 ± 1.90	3.60 ± 1.94	5.934*	0.083	3.40 ± 1.92	3.58 ± 1.92	7.137*	0.094
NPD	3.65 ± 2.21	3.68 ± 2.25	0.178	0.013	3.64 ± 2.28	3.68 ± 2.20	0.398	0.018

Note: *P < 0.05.

## Discussion

The present study indicated that, overall, boys showed more evidence of paranoid, schizotypal, antisocial, and narcissistic PDs than girls, although the evidence are insufficient for the effect sizes are not large enough. This result is consistent with prior reports by Huang et al. [22], Fu and Yao [20], and Fu et al. [21]. We also found that several PD traits (schizoid, borderline, and antisocial) were observed more in freshman and sophomores than in juniors. This grade (age) effect is, to a certain extent, consistent with Johnson et al.’s findings form a community-based longitudinal investigation that PD traits tend to decline steadily in prevalence with advancing age during adolescence and early adulthood [12].

Family structure may also be related to personality pathology. Students from single-parent families scored higher than students from double-parent families on the schizotypal and antisocial PDs, whereas students from remarried families scored higher than students from double-parent families on the borderline and antisocial PDs. Additionally, singleton youths might get higher scores than kids with siblings on the paranoid and antisocial PDs with a insignificant effect. Our family structure effect findings in high school students confirm Huang et al.’s findings in college students in part—like us, they also found that singleton children scored higher on the paranoid and antisocial PDs and that children from single-parent households scored higher on the schizotypal PD. However, our findings differed from Huang et al.’s findings in several ways. For example, they did not see higher antisocial subscores in single-parent versus double-parent households as we did [22]. Huang et al. additionally found significantly lower schizoid subscores for singleton children, versus children with siblings, which we did not find [22]. There are multiple factors that may have contributed to these differences, including possible contributions of differences in familial social and economic statuses between the groups. Additionally, the timing and length of fathers’ absences should be considered when researching PD development in boys [25].

The prevalence of PDs has been reported previously to be inversely related to family socioeconomic factors, such as annual income [26], neighborhood [22], occupational status [27], and employment status [28]. The present findings further support the notion that socioeconomic factors may influence risk of PD development. We found that students who had a low subjective perception of social status in the society ladder of the SSSy scored higher on the schizoid and borderline PDs, but scored lower on the histrionic PD, than students with a high perceived society ladder status. Furthermore, students with a low subjective social status in the school community ladder scored higher than students with a high subjective social status in the school community ladder on the paranoid, schizoid, borderline, and antisocial PDs, while scoring lower on the histrionic PD. Goodman et al.’s research showed that younger adolescents had higher perceptions of social status in society than older adolescents, although age was not significantly associated with adolescents’ responses for the school community ladder [23]. Therefore, as adolescents mature, the influence of subjective social status in the school community ladder on their personality development becomes greater than the influence of subjective social status in the society ladder.

There are some important limitations of this study that should be mentioned. First, although we did not make formal diagnoses, the PDQ-4+ instrument can be strongly over-inclusive when the clinical significance scale is not used. Further studies involving clinical interviews should be conducted to improve the reliability of the questionnaire score. Second, because we conducted a cross-sectional study, not a longitudinal study, these data cannot be used to make claims on direct connections between PDs in adolescence and adulthood. However, we know from other studies that PDs in adolescence can have a profound impact on associated personality traits in adulthood [11,12]. Longitudinal research is necessary to further explicate the possibility that PDQ-4+ scores in adolescence may predict personality pathology in adulthood. Third, although several statistical significances were found by *p* values less than 0.05, all effect sizes are weak which may have a certain impact on the reliability of the conclusion inference. Further clinical studies on personality disorders patients need to be conducted to assess the relationship between personality disorders and demographic variables. Finally, because this study is part of a larger project examining Chinese adolescents’ risk behaviors and related factors, we focused on cluster A and cluster B PD traits only. This limitation of scope allowed us to have greater focus on the areas of interest for our larger project, and thus improved the quality of results within this scope. However, the trade-off for this focus was that we did not examine cluster C PDs (avoidant, dependent, obsessive-compulsive) or other PD behaviors, such as passive-aggressive and depressive traits, although such features are observed in adolescents. To obtain a full understanding of PD characteristics in adolescence and their relationships to demographic variables, it will be important clinically to have data on all PDs.

## Conclusion

The present cross-sectional study revealed significant differences in the presence of cluster A and cluster B PD traits between Chinese adolescents groups in relation to gender, age, family structure, and perceived social status. Studies with a longitudinal design and studies examining the full scope of PDs are needed to fully elucidate the impact of demographic variables on the prevalence of PDs and the clinical relevance of PD traits in adolescence on PDs in adulthood.

## Competing interests

The authors declare that they have no competing interest.

## Authors’ contributions

XZ, JY and SY partially participated in the design of the study. YW, LC, QW, MW were involved in the data collection. YW and XZ performed the statistical analysis and draft the manuscript. All authors read and approved the final manuscript.

## Pre-publication history

The pre-publication history for this paper can be accessed here:

http://www.biomedcentral.com/1471-244X/13/116/prepub
